# Effectiveness of registered nurses on system outcomes in primary care: a systematic review

**DOI:** 10.1186/s12913-022-07662-7

**Published:** 2022-04-04

**Authors:** Julia Lukewich, Shabnam Asghari, Emily Gard Marshall, Maria Mathews, Michelle Swab, Joan Tranmer, Denise Bryant-Lukosius, Ruth Martin-Misener, Allison A. Norful, Dana Ryan, Marie-Eve Poitras

**Affiliations:** 1 Faculty of Nursing, Memorial University, 300 Prince Phillip Drive, St. John’s, NL A1B 3V6 Canada; 2 Department of Family Medicine, Memorial University, 300 Prince Phillip Drive, St. John’s, NL A1B 3V6 Canada; 3 Department of Family Medicine Primary Care Research Unit, Dalhousie University, 1465 Brenton Street, Suite 402, Halifax, NS B3J 3T4 Canada; 4 Department of Family Medicine, Schulich School of Medicine & Dentistry, University of Western, Ontario 1151 Richmond Street, London, ON N6A 5C1 Canada; 5 Health Sciences Library, Faculty of Medicine, Memorial University, 300 Prince Phillip Drive, St. John’s, NL A1B 3V6 Canada; 6 School of Nursing, Queen’s University, 92 Barrie Street, Kingston, ON K7L 3N6 Canada; 7 School of Nursing, McMaster University, 1280 Main St W, Hamilton, ON L8S 4L8 Canada; 8 School of Nursing, Dalhousie University, 5869 University Ave. St, Halifax, NS B3H 4R2 Canada; 9 School of Nursing, Columbia University, 630 West 168th Street, New York, NY 10032 USA; 10 Département de médecine de famille et médecine d’urgence, Université de Sherbrooke, 2500 Boulevard de l’Université, Sherbrooke, QC J1K 2R1 Canada

**Keywords:** Effectiveness, Primary healthcare, Registered nurse, Primary care nursing, Systematic review, Outcomes, Care delivery

## Abstract

**Background:**

Internationally, policy-makers and health administrators are seeking evidence to inform further integration and optimal utilization of registered nurses (RNs) within primary care teams. Although existing literature provides some information regarding RN contributions, further evidence on the impact of RNs towards quality and cost of care is necessary to demonstrate the contribution of this role on health system outcomes. In this study we synthesize international evidence on the effectiveness of RNs on care delivery and system-level outcomes in primary care.

**Methods:**

A systematic review was conducted in accordance with Joanna Briggs Institute methodology. Searches were conducted in CINAHL, MEDLINE Complete, PsycINFO, and Embase for published literature and ProQuest Dissertations and Theses and MedNar for unpublished literature between 2019 and 2022 using relevant subject headings and keywords. Additional literature was identified through Google Scholar, websites, and reference lists of included articles. Studies were included if they measured effectiveness of a RN-led intervention (i.e., any care/activity performed by a primary care RN within the context of an independent or interdependent role) and reported outcomes of these interventions. Included studies were published in English; no date or location restrictions were applied. Risk of bias was assessed using the Integrated Quality Criteria for Review of Multiple Study Designs tool. Due to the heterogeneity of included studies, a narrative synthesis was undertaken.

**Results:**

Seventeen articles were eligible for inclusion, with 11 examining system outcomes (e.g., cost, workload) and 15 reporting on outcomes related to care delivery (e.g., illness management, quality of smoking cessation support). The studies suggest that RN-led care may have an impact on outcomes, specifically in relation to the provision of medication management, patient triage, chronic disease management, sexual health, routine preventative care, health promotion/education, and self-management interventions (e.g. smoking cessation support).

**Conclusions:**

The findings suggest that primary care RNs impact the delivery of quality primary care, and that RN-led care may complement and potentially enhance primary care delivered by other primary care providers. Ongoing evaluation in this area is important to further refine nursing scope of practice policy, determine the impact of RN-led care on outcomes, and inform improvements to primary care infrastructure and systems management to meet care needs.

**Protocol registration ID:**

PROSPERO: International prospective register of systematic reviews. 2018. ID=CRD42018090767.

**Supplementary Information:**

The online version contains supplementary material available at 10.1186/s12913-022-07662-7.

## Background

Primary care providers are the first contact and principal point of continuing care for patients within the healthcare system, and coordinate other specialist care and services that patients may need [1, 2]. Primary care is commonly delivered in an office or clinic setting, with increasing virtual care options, by a team of healthcare providers that often include family physicians working alongside registered nurses (RNs), nurse practitioners, physician assistants, social workers, dieticians, or pharmacists [3, 4]. Team-based primary care, which is the delivery of health services by at least two healthcare providers who work collaboratively to accomplish shared goals with patients/caregivers, has the potential to improve quality, comprehensiveness, coordination, and effectiveness of care, as well as patient and provider satisfaction [5, 6]. The collaborative relationship between physicians and RNs is a key component in the delivery of primary care, with physician/RN teams well-positioned to influence positive outcomes for patients, families, and the healthcare system [7, 8].

Internationally, the primary care RN workforce is growing, but at a different pace across countries [9, 10]. In Australia, primary care nurse employment is increasing the fastest, with 63% of general practices employing a primary care nurse (82% of which are RNs) [11, 12]. In Canada, RNs make up about 70% of the primary care/community health nursing workforce [13]. Typically, RNs have completed either a college diploma or a baccalaureate degree and are able to care for patients with complex health needs who have unpredictable health outcomes. RNs have a more narrow scope of practice than nurse practitioners, and a wider scope of practice than licensed practical nurses (known as registered practical nurses in Ontario) [14]. In primary care settings, RNs function as generalists and provide a broad range of patient services, including preventative screening, health education and promotion, chronic disease prevention and management, acute episodic care, and a wide variety of therapeutic interventions [15–18]. Although job titles used to refer to RNs in primary care vary across countries, common titles include ‘family practice nurse’, ‘primary care nurse’, ‘general practice nurse’, and ‘primary health care nurse’ [19]. For the purpose of this paper, the term ‘primary care RN’ will be used hereafter when referring to this role. Internationally, policy-makers and health administrators are seeking evidence to inform further integration and optimal utilization of RNs within primary care teams [20, 21].

Recently, a systematic review conducted by Norful et al. [17] synthesized international literature related to primary care RNs and made recommendations for optimizing their roles within team-based primary care settings. This review included 18 studies from eight countries. Assessment, monitoring, and follow-up of patients with chronic diseases were identified as fundamental roles of the primary care RN [17]. In addition, countries such as Australia, Canada, New Zealand, and the United Kingdom have developed national standards of practice or defined competencies to articulate the unique roles of primary care RNs [13, 22–26]. Overall, the roles and activities of primary care RNs are becoming increasingly explored and understood internationally. However, the body of literature examining RN effectiveness in the primary care setting has not yet been synthesized. In general, research examining RN effectiveness has primarily been conducted within the acute care setting and focused on staffing, role enactment, and work environment. Within acute care, there is substantial evidence demonstrating the positive effects of the RN workforce on reducing adverse patient outcomes [27–29]. The ongoing evaluation and reporting of care delivered by primary care RNs is important to further refine nursing scope of practice policy, determine the impact of RN-led care on outcomes, and inform primary care infrastructure and systems management.

### Theoretical foundation

The Nursing Role Effectiveness Model offers a framework to guide research examining nursing effectiveness (see Supplementary file 1). This model was developed based on the 1966 Donabedian [30] structure-process-outcome model of quality care and a literature review on nursing-sensitive outcomes and effectiveness of nursing interventions [31]. The structure component of the model consists of patient, nurse, and organizational variables that influence the roles and activities of RNs and outcomes of care [31]. The process component is focused exclusively on nursing interventions, which are treatments, procedures, or roles and actions that the nurse performs to enhance the patient’s health status or behaviour to move towards a desired outcome [32, 33]. The process component describes nurse activities according to three categories: independent, dependent, and interdependent [31, 34–37]. Independent roles are enacted by nurses autonomously, without physician oversight, and typically include assessment and surveillance (e.g., pain), triage, health promotion, risk factor screening, and the implementation of nursing interventions. In contrast, dependent roles describe activities that are part of an expanded nursing scope of practice and are conducted in response to physician medical orders, such as the implementation of medical treatments and prescribing of medications. Interdependent roles are activities nurses share with other members of the healthcare team, such as communication, consultations with other providers, and coordination of care. The Nursing Role Effectiveness Model allows for the conceptualization of the nursing contribution to outcomes of care, namely, functional health outcomes (e.g., physical, social, cognitive, mental functioning), self-care abilities, clinical outcomes (e.g., symptom control and management), prevention of adverse events (e.g., injury or nosocomial infections), patient’s knowledge and engagement (e.g., disease, treatments, management), patient satisfaction, and cost. A scoping review synthesized literature that has used the Nursing Role Effectiveness Model in all healthcare sectors to explore the applicability of using the model in primary care [37]. This review identified 22 articles that applied the model within their research framework. Eighteen of these studies were conducted in Canada or the United States, and 12 studies were focused on the acute care setting. To date, no known research has utilized this model to guide the evaluation of primary care RNs.

### Purpose

Although existing literature provides some information about the contributions of RNs towards outcomes of care, a systematic review synthesizing the effectiveness of this important and growing role within team-based primary care settings is needed. The Cochrane Database of Systematic Reviews, the Joanna Briggs Institute (JBI) Library of Systematic Reviews, and the Prospective Register of Systematic Reviews (PROSPERO) were searched prior to commencement of this study and no registered protocols or previous systematic reviews on this topic were identified. Synthesizing evidence of primary care RNs on quality and cost of care is necessary to demonstrate the contribution of this nursing role and to inform decisions and policies that support the implementation and optimization of primary care RNs going forward [38, 39]. The purpose of this systematic review is to synthesize international evidence on care delivery and system outcomes of primary care RNs to support future best practices in care and research in this field.

## Methods

### Design

A systematic review was conducted using JBI Systematic Review Methodology [40] and findings were reported in accordance with the Preferred Reporting Items for Systematic Reviews and Meta-Analysis (PRISMA) framework [41, 42] (the 2021 PRISMA guidelines were applied where possible). A systematic review approach was selected, given its utility for analyzing and synthesizing literature and evaluating outcomes [43]. Throughout each step of the review, Covidence software was used to efficiently manage and organize the literature [44] and enable a team approach for study and data review. The protocol for this systematic review is registered on PROSPERO (registration ID CRD42018090767). This paper presents findings from studies that report on care delivery and system outcomes. Findings from studies that measured patient outcomes are reported in the companion paper *“Effectiveness of Registered Nurses on Patient Outcomes in Primary Care: A Systematic Review”* [45].

### Search strategy

The search strategy aimed to include both published and unpublished literature. A limited search of CINAHL and MEDLINE databases were conducted initially to identify optimal search terms and keywords by examining subject headings, titles, abstracts, and index terms of similar articles. Using identified targeted keywords and controlled vocabulary, we performed a comprehensive search of relevant electronic databases and grey literature (see Supplementary file 2). Applicable subject headings and keywords (e.g., “primary care”, “registered nurse”, “family practice”) were searched in CINAHL, MEDLINE Complete, PsycINFO (via EBSCOhost), and Embase (via embase.com) for published literature and ProQuest Dissertations and Theses and MedNar for unpublished literature. Unpublished literature was also identified using Google Scholar and the websites of relevant nursing organizations, such as the International Nursing Council, Canadian Family Practice Nurses Association, and Community Health Nurses of Canada. Reference lists of included articles were also searched to identify any additional studies. Database searches were conducted in January, 2019 and January, 2022 by a health sciences librarian (member of the study team); ongoing searches for grey literature included studies with publication dates up to January, 2022. Searches were limited to English-language citations, and no date limiters were applied.

### Inclusion and exclusion criteria

The following pre-established article selection criteria were applied to the search strategy and screening process.

*Inclusion criteria:*

- Studies that focused on RNs or equivalent. A recently completed review of international literature identified regulatory terms used to describe RNs working in primary care [19].
- Studies that were conducted in a primary care setting.
- Studies that measured outcomes attributable to a RN intervention.
- Studies that used any quantitative design (e.g. randomized controlled trial, controlled before-after)
- Studies that were published in English.

*Exclusion criteria:*

- Studies that focused on advanced practice nurses, such as nurse practitioners.
- Studies that did not specify regulatory nursing designation (e.g., referred to nursing in general).
- Studies that were conducted in a setting other than primary care (e.g., acute care, specialist’s office)
- Studies that did not examine a RN-led intervention (e.g., examined outcomes related to structural variables, such as staffing of RNs, in a practice).
- Studies that required RNs to undergo considerable training in a particular area that went beyond the scope of generalist primary care practice (e.g., advanced training in the management of a specific disease, such as COPD).

According to the Nursing Role Effectiveness Model, nursing interventions are defined as “those that are relevant, based on nurses’ scope and domain of practice and for which there is empirical evidence linking nursing inputs and interventions to the outcomes” [47]. Outcomes of interest included, but were not limited to, those identified within the Nursing Role Effectiveness Model (e.g., functional status, patient satisfaction, cost, occurrence of adverse events such as falls or hospitalizations, clinical outcomes such as symptom frequency and severity) [31, 36, 37].

### Screening

Prior to the title/abstract and full-text screening, an eligibility tool was developed by the research team outlining specific inclusion/exclusion criteria. A pilot screening was then conducted amongst three members of the research team, in which the same subset of titles/abstracts and full-text articles were screened independently. Discrepancies amongst reviewers were then discussed and the inclusion/exclusion tool was refined to increase clarity of the selection criteria. Based on best practice recommendations for systematic review screening, this process was repeated until all research team members applied the screening criteria consistently [46].

Covidence software facilitated a collaborative team approach to screening in which two authors (DR and JL) and two trained research assistants were involved. Following the initial pilot testing, all identified titles/abstracts were screened independently by two reviewers for potential study eligibility. Two reviewers then independently retrieved and screened full-text articles for relevancy, applying pre-established eligibility criteria. Any disagreements were resolved through discussion, or by a third reviewer.

### Risk of bias

The risk of bias and quality of each study was assessed using the *Integrated Quality Criteria for Review of Multiple Study Designs* (ICROMS) tool [48], which is a comprehensive multi-design quality appraisal instrument. The ICROMS tool includes a list of quality criteria specific to each study design as well as a ‘decision matrix’, which specifies the minimum threshold that each study design needs to reach in order to be considered acceptably robust. The ICROMS tool scoring matrix was used to determine a quality score for each article (see scoring matrix located in Supplementary file 3). Following a pilot test, in which reviewers initially appraised 2–3 articles to increase comprehension of the tool and resolve any differences in assessment approaches, all full-text articles that met eligibility criteria were appraised for quality by two independent reviewers. The final scores were compared and discussed between both reviewers. Consensus on a final score was considered when both reviewers rated the quality within 2 points in either direction on the scoring matrix. All studies that met inclusion/exclusion criteria also met the minimum ICROMS score to be included in the review.

### Data extraction and synthesis

All eligible full-text studies that met quality criteria underwent a data extraction procedure. The data extraction tool was designed prior to the start of the review by the research team and based on the Cochrane Public Health Group Data Extraction Template [49]. Two articles were selected at random and used to pilot test the tool by three members of the research team, during which time suggestions and alterations were made and a final draft was agreed upon. Data extracted from the articles included: country and year of publication, study aim, design, description of primary care setting, sample size, patient demographics, details of study intervention, RN involvement/role, description of outcome measures/data collection tools, and study results. Due to the heterogeneity of included studies, such as different methodologic approaches, study populations, interventions, and outcome measures, studies were synthesized in narrative format and studies that reported on similar outcomes were grouped together. To address the broad range of terms and descriptors used across included studies, (e.g., traditional care, standard care, basic support, care delivered by anyone other than a primary care RN), and to provide clarity in the presentation of our results, we refer to all control groups as “usual care” or the “comparator group”.

## Results

**Fig. 1:**
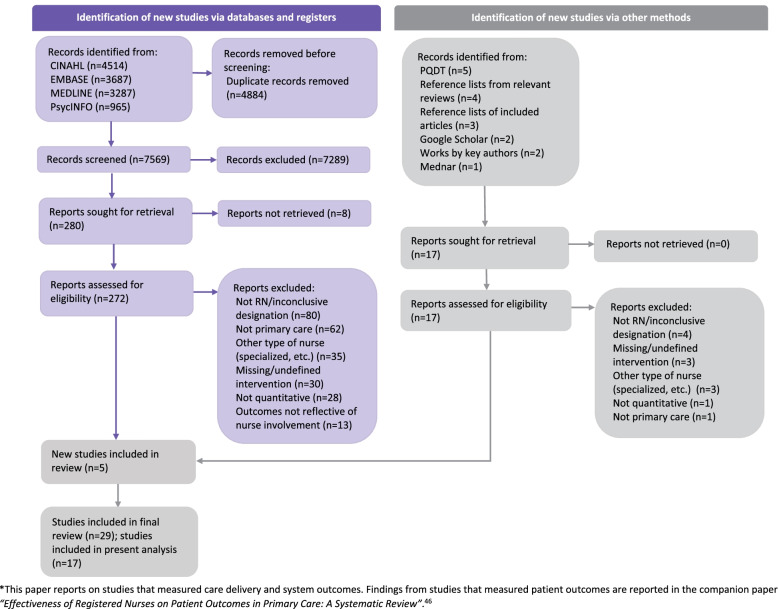
PRISMA Diagram of Literature Search

After removal of duplicate articles, a total of 13,977 published titles and abstracts retrieved from database sources and 17 articles retrieved from grey literature sources were screened for relevancy, resulting in 272 full-text articles from database sources and 17 full-text articles from grey literature sources to undergo assessment by two independent reviewers. Following screening for eligibility and quality appraisal, data were extracted from a total of 29 studies, which were included in the final review (studies were only excluded based on eligibility criteria; none were excluded due to low quality). Fig. 1 presents a PRISMA diagram outlining the results of the literature search.

### Study characteristics

Of the 29 articles included in the final review, 17 reported on care delivery and system outcomes (included in the present analysis) [45]. Table 1 presents a detailed summary of the study characteristics for each article reporting on care delivery and system outcomes (*n* = 17). Studies were published between the years 1996–2021. The majority of the studies were conducted in the United Kingdom (*n* = 8) and the United States (*n* = 5), with the remaining studies originating from Australia (*n* = 2) and New Zealand (*n* = 2). Study designs included randomized controlled trials (*n* = 9), quasi-experimental (no control/comparator group) (*n* = 5) (e.g., survey, cost-analysis), cohort (*n* = 1), non-controlled before-after (*n* = 1), and a mixed-methods design that included both quasi-experimental and non-controlled before-after (*n* = 1). Sample sizes ranged from 126 to 1906 patients. Quality scores, as assessed by the ICROMS tool, varied between studies. Three studies were scored at the minimum threshold for their study design [51, 57, 60], three studies scored 1–2 points above threshold [52, 53, 65], and eleven studies exceeded the minimum cut-off score by 3 or more points [50, 54–56, 58, 59, 61–64, 66].

**Table 1:** Literature Review Table of Study Characteristics (*n* = 17)

Author, Year, Country	Aim	Design	Sample	Intervention and Primary Care RN Involvement	Primary Care Setting Type	ICROMS Quality Appraisal Score^**1**^
Aubert et al., 1998 [50] USA	To compare diabetes control in patients receiving nurse case management and patients receiving usual diabetes management in a primary care setting	Randomized controlled trial	Prudential HealthCare health maintenance organization members with diabetes (*n* = 138 patients were randomized, *n* = 100 provided 12-month follow-up data)	Nurse case management for patient diabetes control (diabetes management delivered by a RN case manager) v. usual diabetes care (control) One RN provided the intervention for this study; RN had 14 years of clinical experience and was a certified diabetes educator RN provided intervention with support- met at least biweekly with the family medicine physician and the endocrinologist to review patient progress, medication adjustments. RN was trained in the delivery of care while primary care providers oversaw clinical decisions.	2 primary care clinics within a group-model health maintenance organization in Jacksonville, Florida	25
Azariah et al., 2013 [51] New Zealand	To increase opportunistic testing for chlamydia in under 25-year-olds and to improve documentation of partner notification in primary care using a nurse-led approach	Uncontrolled before-after	All sexually active under 25-year-olds (*n* = 760) that consented to a chlamydia test during the 4-month pilot project period	Chlamydia management guidelines regarding opportunistic testing and partner notification (analysis of laboratory testing data and diagnostic information) compared to pre-study testing levels Number of RNs and additional characteristics were not indicated RN provided intervention independently. During routine patient visits, RNs recommended chlamydia tests to all patients meeting study criteria; those that tested positive were recalled by the RN for treatment and to discuss partner notification. RNs then provided follow-up call one week after treatment	10 primary care practices in urban South Auckland that operate by a nurse-triage process	22
Bellary et al., 2008 [52] UK	To investigate the effectiveness of a culturally sensitive, enhanced care package for improvement of cardiovascular risk factors in patients of South Asian origin with type 2 diabetes	Cluster randomized controlled trial	Adult patients of South Asian origin with type 2 diabetes (*n* = 1486)	Enhanced management care for type 2 diabetes tailored to the needs of the South Asian community (enhanced care [additional time with PN + support with link worker and diabetes-specialist nurse]) v. standard care [routine PN-led diabetes clinics guided by prescribing algorithm] (control) Number of PNs not indicated; all were formally trained in diabetes management PN provided intervention with support of diabetes nurse specialist, link worker and physician. PNs worked with primary care physicians to implement the protocol and encourage appropriate prescribing, provide patient education, and achieve health targets	21 inner-city practices in 2 cities in the UK with a high-population of South Asian patients. Patients were randomly allotted to the intervention or the control group between March 2004–April 2005	24
Cherkin et al., 1996 [53] USA	To evaluate the impact of a proactive and patient-centered educational intervention for low back pain involving a nurse-intervention group in comparison with two lower impact treatment models	Randomized controlled trial	Patients aged 20–69 years visiting the clinic for back pain, low back pain, hip pain or sciatica (*n* = 294) were randomly allocated to one of 3 groups; *n* = 286 provided complete follow-up data	Educational intervention for back pain carried out by a RN. Usual care (control) v. usual care + educational booklet (booklet intervention) v. usual care + session with RN + educational booklet (nurse intervention); outcomes assessed at 1, 3, 7, and 52 weeks Study involved 6 female RNs with at least 20 years of clinical experience. Study RNs received 9 h of training on the management of back pain RN provided intervention indepdendently. The intervention involved a 15–20-min educational session, including the booklet and a follow-up telephone call 1–3 days later	Suburban primary care clinic in western Washington state, belonging to a staff model Health Maintenance Organization	24
Daly et al., 2000 [54] New Zealand	To evaluate trends in foot examinations for people with diabetes by PNs, district nurses, and specialized nurses between 2006–2008 and 2016 and to determine whether the diabetes education of nurses is related to their management of foot diseases	Quasi-experimental; two cross-sectional surveys	287 randomly selected PNs were surveyed in 2006–2008 and 336 PNs were surveyed in 2016. Nurses provided consulting information for 265 and 166 patients for 2006–2008 and 2016, respectively.	PN-provided examinations and education provision for patients with diabetes foot disease in 2006–2008 and in 2016 Survey was completed by 210 PNs in 2006–2008 and 274 PNs in 2016. Level of diabetes education and years since graduation varied No specific nurse intervention; study examined PN activities and assessments routinely performed during a diabetes consultation on a randomly selected day. Nurses who had consulted at least one person with diabetes on this day were asked about assessments and care provided for these patients, specifically in regards to foot care	General practices that employed a PN across three district health boards in Auckland, NZ	21
Farford et al., 2021 [55] USA	To evaluate the impact of a RN-led Medicare annual wellness visit on preventive services in a family medicine clinic	Quasi-experimental; retrospective chart review	A total of 630 patients (330 undergoing annual wellness visits and 300 undergoing standard assessments) aged 68–72 years who were Medicare beneficiaries	RN-led annual wellness visits of Medicare beneficiaries compared to standard assessment (defined as a 30-min office visit with the primary care physician) The annual wellness visits were conducted by 3 care team RNs (one at each location). Each RN received a 1-h lecture on annual wellness visits and was required to observe a previously trained RN perform annual wellness visits on live patients before offering the service RNs provided intervention independently; during the annual wellness visits, the RNs ordered needed preventive imaging, labs, and vaccinations. The preventive services that were evaluated included mammography, colon cancer screening, bone mineral analysis, pneumococcal vaccination, influenza vaccination, screening for hepatitis C, and screening for depression in a 12-month period.	3 satellite community-based practices connected with the Department of Family Medicine at the Mayo Clinic in Florida	19
Faulkner et al., 2016 [56] UK	To compare differences in smoking cessation treatment delivered by PNs or HCAs on short and long-term abstinence rates from smoking	Cohort study using longitudinal data from a previously conducted randomized controlled trial	Current smokers aged 18–75 years who are fluent in English, not enrolled in another formal smoking cessation study or program and not using smoking cessation medications (*n* = 602)	Smoking cessation support provided by PNs v. HCAs, to compare and assess effects on short and long-term smoking abstinence rates on patients Number of PNs and additional characteristics were not indicated PNs provided intervention alone (and were compared to same intervention provided by HCAs). Patients in both groups received an initial consultation, followed by a program-generated cessation advise report tailored to the smoker and a 3-month program of tailored text messages sent to their mobile phone	32 general practices in East England; 8 of which were in the top 50% of deprived small geographical areas in England	21
Gallagher et al., 1998 [57] UK	To determine the impact of telephone triage, conducted by a PN, on the management of same day consultations in a general practice	Quasi-experimental (cross-sectional) and uncontrolled before-after using prospective telephone and practice consultation data + patient postal questionnaire	All patients in practice (*n* = 1250 consultations with diagnosis), in which consultations were recorded between August 1995–October 1995	Nurse operated telephone consultations/ triage There was a total of 4 PNs working in the practice; the telephone consultation/triage service was managed by a single nurse who had 15 years of experience and was familiar with managing acute illnesses and conducting telephone consultations PN provided intervention with support of physician and receptionist. Patients who telephoned requesting to see a doctor on the same day were put through to the PN, where they would manage the patient’s problem over the phone or arrange for a same-day appointment with either themselves or the GP	Individual general practice in an urban city in England that contains physicians, practice nurses and admin staff	16 22^a^
Harris et al., 2015 [58] UK	To determine whether a primary care nurse-delivered complex intervention increased objectively measured step-counts and MVPA when compared to usual care	Cluster randomized controlled trial	60–75-year-olds who could walk outside and had no contraindications to increasing physical activity (*n* = 298 patients from *n* = 250 households) were recruited between 2011 and 2012 from a random sample of eligible households	Individually-tailored PN consultations centered around physical activity (four physical activity consultations with nurse) v. usual care (no trial contacts other than for data collection at baseline, 3 months and 12 months) (control) Number of PNs and additional characteristics were not indicated PN provided intervention alone; physical activity consultations incorporated behavioural change techniques, step-count and accelerometer feedback, and an individual physical activity plan	3 general practices located in Oxfordshire and Berkshire, UK	28
Harris et al., 2017 [59] UK	To evaluate and compare the effectiveness of pedometer-based and nurse-supported interventions v. postal delivery intervention or usual care on objectively measured physical activity in predominantly inactive primary care patients	Cluster randomized controlled trial	A random sample of 45–75-year-olds without contraindications to increasing MVPA (*n* = 956 with at least one follow-up) were sent postal invitations between September 2012–October 2013	Nurse-supported individually-tailored physical activity consultations as measured by patient pedometer activity (nurse-supported pedometer intervention [arm 1]) v. postal pedometer intervention (arm 2) v. usual care (control) Number of PNs and additional characteristics were not indicated PN provided intervention alone; nurse-supported intervention group involved a pedometer, patient handbook, physical activity diary, and three individually tailored PN consultations offered at 1, 5, and 9 weeks	7 general family practices with an ethnically and socioeconomically diverse population in South London	26
Iles et al., 2014 [60] Australia	To determine the economic feasibility of using a PN-led care model of chronic disease management in Australian general practices in comparison to GP-led care	Randomized controlled trial; cost-analysis	Patients > 18 years of age with one or more stable chronic diseases (type 2 diabetes, ischemic heart disease, hypertension) (*n* = 254)	PN-led care model of chronic disease management v. GP-led care model (usual care) There were 2 PNs and 1–4 GPs involved in each practice over the 2-year study period PN provided intervention alone, working within their scope of practice and from protocols, rather than under supervision of GP; if patients in the PN-led group became unstable, they could be referred back to the GP-led group until their health re-stabilized	3 general practices (urban, regional, rural)	22
Karnon et al., 2013 [61] Australia	To conduct a risk adjusted cost-effectiveness analysis of alternative applied models of primary health care for management of obese adult patients based on level of practice nurse involvement (high-level PN practice v. low-level PN practice v. physician-only model)	Quasi-experimental; risk-adjusted cost-effectiveness analysis	Patients with BMI < 30 prior to October 1, 2009, had at least three visits within the last 2 years, at least two recorded measures of BMI, and aged 18–75 years (*n* = 383 patients were recruited, *n* = 208 were excluded, *n* = 150 patients included in the analysis) who gave consent for researchers to access their medical data	PN involvement in the provision of clinical-based obesity care. Models of care classification were based on percentage of time spent on clinical activities: high-level model (*n* = 4), low-level model (*n* = 6), physician-only model (*n* = 5; due to low number of eligible patients in the physician-only model, data were not presented) Number of PNs were not indicated, although results suggest that high-level practices had a non-significantly higher number of full-time equivalent PNs than low-level practices (0.35 to 0.25, *p* = 0.34); PNs had varying scopes of practice in clinics, which was informed by survey responses that assessed their clinical-based activities No specific nurse intervention; study examined nursing care related to obesity in general (e.g., education, self-management advice, monitoring clinical progress, assessing treatment adherence)	15 of 66 general practices within the Adelaide Northern Division of General Practice with varying levels of PN involvement	22
Katz et al., 2004 [62] USA	To compare medical assistants’ and LPNs’ performance of recommended smoking-cessation guidelines with that of RNs	Secondary analysis of data from a randomized controlled trial	Patients aged 18+ years who had an appointment with a primary care provider for routine care, and reported smoking at least one cigarette per day on average (*n* = 1221)	Smoking cessation clinical practice guidelines implemented by either medical assistants, LPNs, or RNs Number of RNs and additional characteristics were not indicated RNs (and other health professionals in the study) were paired with a primary care clinician but provided the intervention alone and separate. Intervention involved using guideline algorithms and motivational interviewing	9 community-based, primary care practices	27
Low et al., 2005 [63] UK	To evaluate the effectiveness of a PN-led strategy to improve the notification and treatment of partners of people with chlamydia infection compared to standardized protocols for patient referral	Randomized controlled trial	Patients who had received a positive chlamydia test result at their general practice (*n* = 140) from March 2001–October 2002	PN-led strategy to improve the notification and treatment of partners of people diagnosed with chlamydia v. standard protocols, which involve referral to a specialist health advisor at a genitourinary clinic (control) Study involved 36 PNs; additional characteristics not identified PNs provided intervention with support of health advisors; PNs carried out partner notification at the time of diagnosis, followed by telephone follow-up by health advisors. The intervention included a partner notification interview, patient partner referral, and advise on sexual health and sexually transmitted infections	27 general practices in the urban cities of Bristol and Birmingham	27
Moher et al., 2001 [64] UK	To assess the effectiveness of three different methods for improving the secondary prevention of coronary heart disease in primary care (audit and feedback; recall to a GP; recall to a nurse clinic)	Pragmatic, unblinded, cluster randomized controlled trial comparing three intervention arms	Patients aged 55–75 years with established coronary heart disease (*n* = 1906) as identified by computer and paper health records were recruited from 1997-1999	Secondary prevention care of patients with coronary heart disease delivered at three levels (i.e., audit and feedback; GP recall; nurse recall) Number of PNs in study unknown- all practices employed at least 1 PN; additional characteristics not identified PN provided intervention with support of the trial’s nurse facilitator, who gave ongoing support to the practices in setting up a recall system for review of patients with coronary heart disease. The nurse recall and GP recall groups employed the same intervention	21 general practices in Warwickshire that employed PNs, but were not already running nurse-led clinics	26
O’Neill et al., 2014 [65] USA	To assess expanded CPS and RN roles by comparing blood pressure case management between CPS and physician-directed RN care in patients with poorly controlled hypertension	Quasi-experimental; non-randomized, retrospective comparison of a natural experiment	Patients that had face-to-face or telephone appointments with a RN case manager for poorly controlled hypertension with either physician-directed or CPS-directed clinical decision making at the index encounter (*n* = 126)	Patient hypertension care delivered interdependently by clinical pharmacy specialist-directed RN case management as an alternative to physician-directed RN case management Number of RNs and additional characteristics were not indicated RN provided intervention with support of either CPs or physician; RNs assessed patients independently and presented the case to either a CPS or a physician, if the hypertension continued to be poorly controlled. The RN communicated any changes in the plan to the patient	A large Midwestern Veteran’s Affairs Medical Center that utilizes team-based care	18
Plummer et al., 2000 [66] UK	To determine the ability of a PN to identify psychiatric morbidity in patients attending their clinics, before the implementation training interventions	Quasi-experimental	All patients in practice aged 16 years and over and not suffering from a disorder causing a cognitive impairment (*n* = 1768 approached, *n* = 1710 completed the survey)	Use of data collection and patient questionnaires to determine the abilities of PNs to identify patient psychological distress levels and psychiatric morbidity during nurse consultations with patients One PN from each practice location took part in the study (*n* = 24); additional nurse characteristics not identified PN provided intervention alone; after initial consultation, patients completed a 12-item questionnaire. PN was asked to rate patient’s level of psychological distress on a scale of 0–4. Level of agreement between patient general health questionnaire classification and PN’s assessment was then assessed	24 general practices randomly selected from 41 practices in South London and Kent	19

^a^Mixed methods study consisting of multiple designs; separate ICROMS quality appraisal scores were generated for each study type; *RN* registered nurse, *PN* practice nurse, *HCA* health care assistant, *GP* general practitioner, *MVPA* moderate to vigorous physical activity, *BMI* body mass index, *LPN* licensed practical nurse, *CPS* clinical pharmacy specialist

### Overview of RN interventions

A variety of independent and interdependent RN interventions were examined across eligible studies. Most focused on some aspect of chronic disease prevention and management (*n* = 7) related to diabetes, coronary heart disease, and obesity [50, 52, 54, 60, 61, 64, 65]. Other RN interventions included smoking cessation support [56, 62], chlamydia screening, partner notification and treatment [51, 63], back pain education and management [53], telephone consultation/triage service [57], assessment of psychological distress [66], consultations aimed at increasing patient physical activity levels [58, 59], annual wellness visits [55], and laboratory monitoring [65]. Despite commonalities in study design and type of intervention delivered, strengths and limitations in scope and methodology varied across studies. Additional information regarding research limitations associated with each study are outlined in Supplementary file 4.

The majority of primary care RNs carried out the interventions independently, without a physician’s order or the support of other healthcare providers to respond to patient needs (*n* = 10) [51, 53–56, 58–60, 64, 66], while others carried out the intervention interdependently in association with other healthcare providers (e.g., physicians, health advisors, research assistant) (*n* = 6) [50, 52, 57, 62, 63, 65]. Another study examined the impact of varying levels of nursing involvement (low-level involvement versus high-level involvement) in general practices on patient obesity outcomes [61].

Of the studies included, five examined a RN-led intervention compared to the same intervention delivered by other healthcare providers [56, 60, 62–64], six studies compared RN-led interventions to ‘usual care’, defined as either care that existed prior to the intervention that did not involve a RN (*n* = 3) [50, 53, 59], or care associated with reduced or alternate levels of RN involvement (*n* = 3) [51, 52, 58], and one study compared a collaborative intervention involving primary care RNs supported by two different types of healthcare providers (clinical pharmacy specialists [CPS] and physicians) [65], where RNs assessed patients independently and presented the patient to either a CPS or a physician if hypertension continued to be poorly controlled). Lastly, five studies examined the effectiveness of a primary care RN intervention using a quasi-experimental design as a means of evaluation (i.e., no comparison group) [54, 55, 57, 61, 66].

### Overview of outcomes

Table 2 presents a list of outcomes measured within included studies. Care delivery outcomes included quality and frequency of assessment and infection/disease screening (e.g., annual wellness visits, diabetic foot examinations, coronary heart disease, psychological disorders/distress, chlamydia), quality of smoking cessation support, appropriateness of laboratory monitoring, and quality of prescriptions issued/modified. System outcomes included cost, adverse health events, health service utilization, and changes in workload. A total of 15 care delivery outcomes (see Table 3) and 11 system outcomes (see Table 4) were identified across included studies.

**Table 2:** List of Outcomes Measured in Included Studies

**Outcomes**	
**System Outcomes**	
Cost [51, 52, 60, 61]	
Workload [53, 60]	
Adverse Events (e.g., hypoglycemia, hospital admissions, emergency room visits, falls) [50, 58, 66]	
Service Utilization (e.g., clinic visitations, repeat consultations for same issue) [53, 63]	
**Care Delivery Outcomes**	
Quality of Assessment and Screening (e.g., heart disease, psychological disorders, chlamydia) [51, 54–57, 64]	
Quality of Smoking Cessation Support [54, 62]	
Chlamydia Case Management (e.g., screening, treatment, partner notification) [51, 56]	
Access to Appropriate Medications (i.e., illness management) [53, 64, 65]	
Laboratory Monitoring [65]

**Table 3:** Literature Review Table – Care Delivery Outcomes

Author, Year, Country	Description of Outcome	Results
**Quality of Assessment and Screening**		
Azariah et al., 2013 [51] New Zealand	Number of chlamydia tests completed	There was a large increase in chlamydia testing, with a high prevalence found in the population tested. During the pilot, there was a 300% increase in the number of chlamydia tests in the target age group from 812 to 2410 and the number of male tests increased by nearly 500%. Nurse-led opportunistic testing for chlamydia in primary care is successful at increasing testing in both males and females.
Daly et al., 2000 [54] New Zealand	Rate of foot examinations and foot care education activities performed by PNs in 2006–2008 and in 2016	Significantly more nurses in 2016 than in 2006–2008 self-reported routinely examining patients’ feet (45% versus 31%) and giving foot-care education (28% versus 13%). District nurses were more likely to conduct foot examinations in 2016; however, PNs were significantly more likely than district nurses and specialist nurses to test sensation (*p* = 0.0005). PNs receiving diabetes education (> 5 h) within the last five years was positively associated with conducting foot examinations and providing recommended foot care education. PNs have significantly expanded their role in managing people with diabetes over the last decade by increasing the number of foot examinations and providing recommended foot-care education.
Farford et al., 2021 [55] USA	Number of preventative services utilized by patients (mammography, colon cancer screening, bone mineral analysis, pneumococcal vaccination, influenza vaccination, screening for hepatitis C, and screening for depression)	Each preventive service was utilized more often by patients in the annual wellness visit group than the standard assessment group (all ORs ≥1.64; all *p*-values ≤0.004). The preventive services with the greatest improvement in the annual wellness visit group compared to standard assessment were depression screening (OR = 4.15; 95% CI: 2.57 to 6.70; *p* < 0.001) and mammogram (OR = 3.87; 95% CI: 2.00 to 7.50; *p* < 0.001). A RN-led Medicare annual wellness visit is an effective way of assisting Medicare beneficiaries in meeting their preventative care needs.
Low et al., 2005 [63] UK	Proportion of index cases with at least one treated sexual partner	Overall, 45% (92/206) of contacts of 140 index cases were considered treated: 65.3% (47/72) of cases seen by a PN and 52.9% (39/68) in the control group had at least one sexual partner treated ((OR = 12.4; 95% CI: 1.8 to 26.5; *p* = 0.087). There was no significant difference between the two treatment arms (risk difference 7.9%; CI: -8.4 to 24.0%). PNs with appropriate training and support from health advisors to carry out telephone follow-up can provide immediate partner notification for community diagnosed chlamydia that is at least as effective as referral to a specialist health advisor at a genitourinary medicine clinic.
Moher et al., 2001 [64] UK	Assessment of heart disease at 18 months based on 3 risk factors: blood pressure, cholesterol and smoking status	The groups differed substantially in the proportions of patients being adequately assessed; the absolute increase in the proportion of patients who received adequate assessment compared with the audit group was 33% (95% CI: 19 to 46%) in the nurse group and 23% (95% CI: 10 to 36%) in the GP recall group. Adequate assessment was higher in the nurse group than the GP recall group (85% v. 76%), but the difference was not significant. The other components of adequate assessment all followed a similar pattern.
Plummer et al., 2000 [66] UK	Level of agreement between PN assessment and General Health Questionnaire classification of psychiatric morbidity	The mean detection rate by PNs when identifying significant distress was 16% (between nurse variation, 0 to 61%). A second analysis, changing the nurse criterion to recognition of distress, increased the mean sensitivity rate to 58% (variation 31 to 84%). There was, however, a statistically significant increase in the OR for nurse identification using either criterion, as the General Health Questionnaire score increases (i.e., a higher proportion of more severe cases were detected). Overall, agreement with the General Health Questionnaire classification was modest, however, as patients’ symptoms become more severe, a higher proportion of cases were identified.
**Quality of Smoking Cessation Support**		
Faulkner et al., 2016 [56] UK	Provision and quality of smoking cessation support as defined by time taken for consultation, pharmacotherapies prescribed, advise delivered, and number and type of interim contacts	There was no statistically significant difference in advice delivered, or types of pharmacotherapies prescribed. Compared with nurses, HCA consultations were longer on average (*p =* 0.002) and made more interim contacts (*p* < 0.001). Nurses and HCAs appear to be equally effective at supporting smoking cessation, however, nurses appear to be able to provide equivalent care with less patient contact.
Katz et al., 2004 [62] USA	Performance of guideline-recommended smoking cessation counseling activities (after adjustment for patient-level covariates, intake clinicians’ characteristics, and study site)	Performance of all guideline-recommended counseling activities were significantly greater for all types of nursing personnel at test v. control sites. Adjusting for patient- and visit-related covariates demonstrated that medical assistants were significantly less likely to assess willingness to quit (OR = 0.4, 95% CI = 0.2 to 0.8; *p* = 0.005) and tended to offer advice and assistance in quitting less often than RNs. Similar findings were observed for LPNs when compared with RNs (OR = 0.5; 95% CI: 0.3 to 1.0; p = 0.03). Although both medical assistants and LPNs showed marked improvements in performance in response to the guideline intervention, patients seen by these intake clinicians were less likely to receive guideline-recommended counseling, compared to those patients seen by RNs.
**Chlamydia Case Management**		
Azariah et al., 2013 [51] New Zealand	Level of documentation of partner notification in diagnosed cases of chlamydia	The pilot resulted in the recording of more information regarding follow-up and outcomes of partner notification in the Patient Management System.
	Number of chlamydia tests completed	There was a large increase in chlamydia testing, with a high prevalence found in the population tested. During the pilot, there was a 300% increase in the number of chlamydia tests in the target age group (812 to 2410) and the number of male tests increased by nearly 500%. Nurse-led opportunistic testing for chlamydia in primary care is successful at increasing testing in both males and females.
Low et al., 2005 [63] UK	Proportion of index cases with at least one treated sexual partner	Overall, 45% (92/206) of contacts of 140 index cases were considered treated: 65.3% (47/72) of cases seen by a PN and 52.9% (39/68) cases in the control group had at least one sexual partner treated (OR = 12.4; 95% CI: 1.8 to 26.5; *p* = 0.087). There was no significant difference between the two treatment arms (risk difference 7.9%; CI: -8.4 to 24.0%). PNs with appropriate training and support from health advisors to carry out telephone follow-up can provide immediate partner notification for community diagnosed chlamydia that is at least as effective as referral to a specialist health advisor at a genitourinary medicine clinic.
**Access to Appropriate Medications (Illness Management)**		
Gallagher et al., 1998 [57] UK	Number of consultations that resulted in the issuing of a prescription	A total of 51% consultations resulted in a prescription (21% telephone consultations, 51% nurse consultations, 66% doctor consultations, and 65% consultations with both nurse and doctor).
Moher et al., 2001 [64] UK	Amount of hypotensive agents, lipid lowering drugs, and antiplatelet drugs prescribed	Prescribing of hypotensive and lipid lowering agents varied little between the nurse recall, GP recall and audit groups. Prescribing of antiplatelet drugs increased in all groups, but at follow-up the nurse recall group had achieved higher levels of prescribing than the audit group (10% more) and the GP recall group (8% more).
O’Neill et al., 2004 [65] USA	Intensification of patient medication levels for hypertension	All patients (*n* = 126) in the study received medication intensification at the index visit, regardless of intervention type.
**Laboratory Monitoring**		
O’Neill et al., 2004 [65] USA	Relevant laboratory monitoring of patients, defined as an issuing of a basic metabolic panel within 4 weeks of initiation or intensification of a diuretic, angiotensin converting enzyme inhibitor, angiotensin receptor blocker, or aldosterone antagonist	Laboratory monitoring within 4 weeks of initiation or intensification of a diuretic, angiotensin converting enzyme inhibitor, angiotensin receptor blocker, or aldosterone antagonist was completed in 7 out of 37 possible cases in the CPS group (19%) and 14 out of 39 possible cases in the physician group (36%; *p* = 0.13).

*PN* practice nurse, *CI* Confidence Interval, *GP* general practitioner, *HCA* health care assistant, *OR* Odds Ratio, *LPN* licensed practical nurse, *RN* registered nurse, *CPS* clinical pharmacy specialist

**Table 4:** Literature Review Table – System Outcomes

Author, Year, Country	Description of Outcome	Results
**Cost**		
Bellary et al., 2008 [52] UK	Economic analysis of net intervention cost (staff salaries, travel and subsistence, equipment, payment to practices, and prescribing) over 2 years	The economic analysis shows that financial investment needed over 2 years did not produce significant enough health-related gain in quality of life to make the nurse-led intervention clearly cost-effective.
Iles et al., 2014 [60] Australia	Total MBS item charges over a 1-year study period	There was an estimated $129 (Australian dollars) mean increase in total MBS item charges over a 1-year period (controlled for age, self-reported quality of life, and geographic location of practice) associated with PN-led care. Based on cost calculations of salaries and expenditures at the time of the study, it was concluded that Medicare reimbursements provide sufficient funding for general practices to employ PNs within limits of workloads
Karnon et al., 2013 [61] Australia	Cost-effectiveness analysis specifically related to primary care, pharmaceutical, and hospital costs	High-level model patients incurred greater primary care and pharmaceutical-related costs, though hospital costs were greater in the low-level model patients. Incrementally, the high-level model gets one additional obese patient to lose weight at an additional cost of $6741, and reduces mean BMI by an additional one point at an additional cost of $563 (upper 95% CI: $1547).
Low et al., 2005 [63] UK	Cost of each intervention strategy per positive chlamydia index case in 2003 sterling prices	The costs of the two strategies were similar in both study arms: £32.55 (95% CI: 31.20 to 33.91) for the PN-led strategy and £32.62 (95% CI: 31.49 to 33.73) for the specialist referral strategy.
**Workload**		
Gallagher et al., 1998 [57] UK	Changes to number of GP and nurse consultations over three-month study period	Doctor workload fell by 54%, from 1522 to 664 consultations, compared with the previous three months. The number of other appointments provided by the nurses fell by 21%, from 1793 to 1415 appointments. Telephone triage of patients who were contacting the clinic for a same-day appointment reduced doctor workload.
Iles et al., 2014 [60] Australia	Frequency of patient visits to GP and PN	The frequency of GP and PN visits varied markedly according to chronic disease. Cardiovascular disease patients in the PN-led care group made more PN visits than the GP-led care group (4.97 v. 3.23; *p* = 0.013), diabetes patients in the PN-led care group had more PN visits than the GP-led care group (13.29 v. 1.63; *p* < 0.001) and hypertension patients in the PN-led care group had marginally more PN visits than the GP-led care group (4.80 v. 3.12; *p* = 0.013). The notion that PN-led model of care would free up GP workload was not supported.
**Adverse Events**		
Aubert et al., 1998 [50] USA	Episodes of severe hypoglycemia; emergency room and hospital admissions	There were no statistically significant differences between nurse case management groups and usual care for adverse events.
Harris et al., 2015 [58] UK	Falls, fractures, sprains, injuries, or any deterioration of health problems already present at 3 and 12 months	There were no between-group differences in number of adverse events at 3 or 12 months.
Harris et al., 2017 [59] UK	Falls, injuries, fractures, cardiovascular events, deaths at 3 and 12 months	Total adverse events did not differ between groups at 3 or 12 months, however, cardiovascular events over 12 months were lower in the intervention groups than in controls (*p* = 0.04).
**Service Utilization**		
Cherkin et al., 1996 [53] USA	Number of back pain-related visits made by patients to family physicians or other providers between the 3, 7, and 52 week evaluations, as well as number of hospitalizations	The proportion of subjects making at least one visit for low back pain and the mean number of visits were similar for all groups at each follow-up interval; the interventions had no impact on health care use.
Gallagher et al., 1998 [57] UK	Repeat consultations to a general practice for the same acute care related problem	Repeat consultations were significantly higher after one week for nurse consultations than doctor consultations (52% v. 37%; 95% CI: 2 to 28%; *p* = 0.02).

*MBS* Medicare Benefits Schedule, *PN* practice nurse, *BMI* body mass index, *GP* general practitioner, *CI* Confidence Interval

### Care delivery outcomes

#### Quality of assessment and screening

Six studies examined the effectiveness of primary care RN-led assessment and screening. Three studies used a quasi-experimental design (no comparison group); one used patient questionnaires to assess the ability of primary care RNs to detect psychological distress [66], another implemented a cross-sectional survey of primary care RNs to evaluate trends in diabetes-related foot examinations [54], and another carried out a retrospective chart review to assess the impact of primary care RNs on preventative services performed during annual wellness visits [55]. Another study compared laboratory testing data before and after an intervention [51], and two conducted a randomized controlled trial in which RN-led care was examined against two comparator groups (i.e., ongoing physician support and usual care for follow-up of cardiovascular disease risk factors) [64] or usual care alone (standard protocol for partner notification after chlamydia diagnosis) [63]. According to these studies, improved assessment and prevention of coronary heart disease risk factors (i.e., blood pressure, cholesterol, smoking status) [64], adequate assessment of psychological distress levels [66], improved management of diabetic foot examinations [54], successful implementation of recommended preventative care services during annual wellness visits [55], and effective screening for sexually transmitted infection (e.g., chlamydia) [51, 63] can be provided by primary care RNs. Primary care RN-led screening for coronary heart disease risk factors was determined to be as effective as screening conducted by physicians (no significant difference found between groups) [64].

#### Quality of smoking cessation support

Two studies examined the quality of smoking cessation support delivered independently by primary care RNs versus medical assistants [56, 62], with one study offering an additional comparison to care provided by licensed practical nurses [62]. A secondary analysis of a previous randomized controlled trial from the United States found that medical assistants and licensed practical nurses were less likely to provide smoking cessation support in accordance with recommended clinical practice guidelines in comparison to primary care RNs. For instance, medical assistants and licensed practical nurses were less likely to assess willingness to quit smoking than primary care RNs (OR = 0.4; 95% CI: 0.2 to 0.8; *p* = 0.005 and OR = 0.5; 95% CI: 0.3 to 1.0; *p* = 0.03, respectively) [62]. A similar cohort study using longitudinal data from the United Kingdom determined that health care assistants took longer in their smoking cessation consultations with patients (24 min versus 21 min; *p* = 0.002) and provided the patient with more interim contacts (2 versus 1 contact; *p* < 0.001) in order to achieve equivalent outcomes. In this study, the type of smoking cessation provider (i.e., RN or health care assistant) seen by participants was not determined at random. While participants in each group had similar characteristics, there may have been unmeasured patient or provider cofounders that impacted findings [56].

#### Chlamydia case management

Azariah et al. [51] conducted an uncontrolled before-after pilot study of independent primary care RN-led opportunistic chlamydia testing in patients under 25-years of age and found improved case management, demonstrated by an increase in documentation of 1 week treatment follow-up and outcomes of partner notification in the Patient Management System. Similarly, a primary care RN-led strategy (with appropriate training) to improve partner notification for community diagnosed chlamydia patients was determined to be equally as effective as referral to a specialist health advisor at a genitourinary medicine clinic (47 versus 36 cases of at least one treated partner; OR = 12.4; 95% CI:-1.8 to 26.5; *p* = 0.087) [63].

#### Appropriate laboratory monitoring

Only one study in the review examined the appropriate ordering and follow-up of laboratory tests [65]. The authors defined appropriate laboratory monitoring as the ordering of a basic metabolic panel within 4 weeks of initiation or intensification of specific antihypertension agents (i.e., diuretics, angiotensin converting enzyme inhibitors, angiotensin receptor blockers, aldosterone antagonists). A non-randomized, retrospective comparison of a natural experiment compared CPS-supported versus physician-supported primary care RN hypertension case management (RNs conducted assessment independently and involved CPS or a physician if hypertension was poorly controlled). Level of adherence to appropriate laboratory monitoring guidelines was assessed through review of laboratory results after the first patient appointment. The results indicated that laboratory monitoring within 4 weeks was completed in 7 out of 37 (19%) possible cases in the CPS-supported group and 14 out of 39 (36%) possible cases in the physician-supported group, with no significant differences between groups (*p* = 0.13). This demonstrates that primary care RN-CPS collaborative care teams can achieve equivalent outcomes to that of RN-physician teams. However, these findings may not accurately reflect the rate of laboratory tests ordered, as patients who were non-adherent to laboratory monitoring recommendations were excluded from the data analysis, limiting generalizability of the results.

#### Access to appropriate medications (illness management)

Three studies explored primary care RN-led or facilitated illness management, specifically with respect to prescription medication strategies [57, 64, 65]. Gallagher et al. [57] determined the impact of telephone triage conducted independently by a primary care RN on the management of same day requests for consultations. Fifty-one percent (*n* = 647/1262) of the consultations resulted in new or changed prescriptions. The authors concluded that primary care RN triage enhanced efficiency of the practice and allowed for timely medication management. Moher et al. [64], using a cluster randomized controlled trial approach, explored the effectiveness of three interventions (audit and feedback, recall to a physician, recall to a primary care RN clinic) for improving secondary preventive care of patients with coronary heart disease. One of the targeted outcomes was the use of hypotensive, lipid lowering, and anti-platelet drug management. Prescribing of hypotensive and lipid lowering medications was similar between groups, however, prescribing of antiplatelet drugs revealed a small significant difference between the primary care RN recall group and the audit group (10% difference; 95% CI: 3 to 17%; *p* = 0.009), and between the primary care RN recall group and the physician recall group (8% difference; 95% CI: 1 to 15%; *p* = 0.031). O’Neill et al. [65], using a retrospective comparison, compared CPS-supported versus physician-supported primary care RN case management on the optimization of medication management for patients with uncontrolled hypertension using data available within existing electronic clinical records (i.e. clinical progress notes). Medication intensification at the index visit was similar between groups (no significant difference), supporting the use of collaborative teams, consisting of either CPS-or physician-supported primary care RN case management.

### System outcomes

#### Adverse events

Three studies examined adverse events in usual care that did not involve care delivered by a RN versus primary care RN-led interventions for diabetes (i.e., randomized controlled trial examining nurse case management for diabetes control) [50] and physical activity (i.e., clustered randomized controlled trial examining a RN-supported pedometer intervention) [58, 59]. Adverse events measured in these studies consisted of falls, injuries, cardiovascular events, episodes of severe hypoglycemia, emergency room visits and hospital admissions, deaths, and any deterioration of a pre-existing health problem. Two studies found no significant differences between the intervention and usual care groups [50, 58], while the third study found no difference in total adverse events at 3 and 12 months, but a significantly lower number of adverse cardiovascular events over the 12-month study period (*p* = 0.04) for the intervention group [59]. All three of these studies examined a unique role of the RN in supporting diabetes and promoting physical activity, limiting the generalizability of these findings to routine primary care practice.

#### Service utilization

A randomized controlled trial evaluating the impact of an educational intervention for low back pain [53] found no difference in the frequency of clinic visitations for patients who received primary care RN-delivered care versus usual care with the provision of an educational booklet (*p* = 0.7) or usual care alone (no educational booklet or primary care RN educational sessions) (*p* = 0.7). A study examining the effectiveness of primary care RN-led telephone triage for patients seeking a same-day appointment found that repeat consultations for the same problem after 1 week were significantly higher for patients who were triaged to primary care RN consultations than physician consultations (52% versus 37%; 95% CI: 2 to 28%; *p* = 0.02) [57]. However, this study did not assess whether the repeat visit indicated that patient problems were dealt with inadequately at triage.

#### Workload

Primary care RN-led telephone triage of patients seeking a same-day appointment reduced physician visits by 54% (1522 to 664) and primary care RN visits by 21% (1793 to 1415) [57]. However, it is unclear whether or not this decrease in workload was attributable to the intervention or seasonality, as the study compared the intervention period with the 3-month period prior to intervention, rather than with a 3-month period from the same season (i.e., summer) in the previous year. A study of a primary care RN-led model of chronic disease management within a general practice found that the primary care RN-led model of care did not significantly decrease the total number of physician visits, as the total visits per patient more than doubled during the intervention period for all three chronic diseases (type 2 diabetes, cardiovascular disease, hypertension), disputing the notion that the RN-led model of care would free up physician workload [60].

#### Cost

Four studies examined costs associated with primary care RN-led interventions. An Australian costing study found that the costs associated with primary care RNs in general practice clinics could be covered by the additional Medicare Benefit Schedule (MBS) billings generated from the primary care RNs [60]. It should be noted that costing studies describe costs of an intervention (i.e., employing primary care RNs) without considering the health effects of the intervention [67, 68].

Cost-effectiveness studies compare costs of an intervention relative to health effects of the intervention [67, 68]. Karnon et al. [61] compared the costs of primary care RN-led obesity interventions in clinics with high versus low-level involvement of primary care RNs in the clinic. The marginal incremental cost of high-level clinics was $563 (95% CI: $123 to $1547) per one point reduction of body mass index (BMI). The high-level clinics produced a statistically significant reduction in mean BMI compared to low-level clinics, but the total reduction in weight was not clinically significant. The study was unable to compare the intervention to usual care. Another cost-effectiveness study, conducted by Low et al. [63], found that the costs and effects (number of sexual partners treated for chlamydia) did not significantly differ for the primary care RN-led intervention versus usual care in reference to rate of partner notification (mean unit cost = £11.72; 95% CI: 10.37 to 13.08 versus £10.86; 95% CI: 9.74 to 11.98, respectively) or for partner treatment (mean unit cost = £32.55; 95% CI: 31.20 to 33.91 versus £32.62; 95% CI: 31.49 to 33.73, respectively).

Cost utility analyses calculate the costs of the intervention relative to the quality of life changes stemming from the effects of the interventions [67, 68]. Bellary et al. [52] calculated the incremental cost-effectiveness ratio of £28,933 per quality-adjusted life-year (QALY) gained and concluded that the cost needed to fund the primary care RN-led culturally sensitive diabetes intervention over a 2-year period did not produce significant improvements in patient quality of life, given the modest or non-significant differences in clinical outcomes. However, this study focused on a specific patient population (i.e., adult patients of South Asian origin with type 2 diabetes) and findings are based on clinical biomarker changes (i.e., blood pressure, total cholesterol) as the sole measurement of patient quality of life, which ignores elements of the patient experience and other measures that contribute to a more fulsome quality of life measurement.

## Discussion

This systematic review presents synthesized evidence on care delivery and system outcomes by primary care RNs. Overall, the findings indicate that primary care RNs have an impact on the delivery of appropriate, high-quality care that meet patient needs and that RN care can be tailored to specific health conditions, including diabetes, sexually transmitted infections, coronary heart disease, and obesity. Similarly, findings demonstrate that primary care RNs can be effective in the implementation of preventative screening services and the promotion of health behaviors, such as smoking cessation consultations and diabetic foot care education. The studies included in this review captured many variables included in the Nursing Role Effectiveness Model, including independent and interdependent interventions and care delivery (e.g., quality of assessment, screening, and disease management) and system outcomes. Prevention of adverse events is an important component of nursing care and includes the promotion of patient safety and freedom from injury/infection [31]. Likewise, cost outcomes identified in the model may include any direct or indirect costs associated with nursing care and nursing interventions (e.g., health service utilization) [31, 35]. These components of the framework were measured in several studies included in this review. The identification of other outcomes not listed in the Nursing Roles Effectiveness Model could potentially inform a modification of the Nursing Role Effectiveness Model tailored specifically to the primary care setting and roles that RNs commonly perform. Furthermore, although the Nursing Role Effectiveness Model served as a guide to map study variables, many studies did not consider the structural component of the model (e.g., nurse characteristics, such as level of education, years of experience, context of care) which may have impacted outcomes observed or did not always describe specific interventions in detail.

The studies suggest that RN-led care may have an impact on care delivery and system outcomes, specifically in relation to the provision of medication management, patient triage, chronic disease prevention and management, treatment of acute illnesses/conditions, educational interventions, sexual health, health promotion, and self-management interventions, such as smoking cessation support and promotion of physical activity. In particular, there is growing literature demonstrating the benefits (e.g., improved access to medications, physician support) of non-physician prescribing, which involves nurses, pharmacists, and physician assistants substituting for physicians in a prescribing role [69–71]. Specifically, RN prescribing is increasingly recognized as an emerging role within primary care [72, 73]. It is also within the current scope of practice of RNs, regardless of their competencies or education level, to recommend over-the-counter medications to alleviate symptoms or treat minor/acute illnesses, suggest and titrate dosages, discuss medication administration routes, educate patients on the side-effects of medication and drug-drug interactions, and perform medication list reviews [69, 74–76]. Weeks et al. [71] conducted a systematic review that assessed outcomes of non-medical prescribing for managing acute and chronic health conditions in primary and secondary care settings compared with medical prescribing (usual care). Twenty-six studies included in the review reported on outcomes related to non-medical prescribing undertaken by nurses in general (but did not differentiate between nursing provider types). Overall, the findings suggested that non-medical prescribers were as effective as usual care medical prescribers, and that regulators and health administrators should explore this expanded role for RNs as an opportunity to improve medication access and address unmet health needs.

Although this study provides preliminary evidence on outcomes of RNs in primary care with regards to medication management, triage, chronic disease management, sexual health, and health promotion/self-management interventions, the included studies did not capture outcomes related to the many other roles/activities performed by RNs within this setting. Primary care RNs function as generalists who provide a broad range of services. Common roles/activities performed by primary care RNs that were not captured in the studies included in this review are therapeutic interventions (e.g., wound care, treatment of infections), pediatric and women’s health, health prevention and public health services (e.g., immunizations), and care/case coordination (nursing surveillance, professional referral, system navigation). Furthermore, while this study provides preliminary evidence on the effectiveness of RN-led interventions in primary care, research demonstrating the long-term impacts of these interventions is lacking. The lack of longitudinal research does not allow for conclusions to be drawn regarding the long-term impacts of RN interventions (e.g., health promotion, nursing surveillance) on patient morbidity and mortality. High-quality longitudinal research involving the use of a cohort design or analysis of large datasets is needed to explore the effectiveness of primary care RNs over time. The absence of large databases capturing nursing interventions is hindering progress in this field of research. Lastly, the overarching competencies (i.e., integrated knowledge, skills, judgement, and attributes) that guide primary care RN practice include leadership, communication, and collaboration and partnership with other healthcare providers; these competencies, which are also represented in the Nursing Role Effectiveness Model, have yet to be evaluated in the primary care RN literature.

There have been few studies to examine the cost of nursing within any professional designation (i.e., RN, nurse practitioner, licensed practical nurse) in primary care internationally. This review identified only four studies that reported on cost outcomes for RNs in primary care, with substantial variability across studies, limiting the ability to make comparisons and draw firm conclusions. The included studies also did not account for context of care or indirect cost savings from RN contributions (e.g., savings in physician costs). In addition, the financial impact and cost reduction associated with long-term health prevention is difficult to measure and capture in the literature because cohort studies are difficult to conduct in a primary care setting. In contrast, there is increased evidence of the added value of nurse practitioners, more specifically improved clinical outcomes and patient and provider satisfaction, through several randomized controlled trials and systematic reviews [77]. However, due to limitations and challenges with economic evaluations, the question of cost-effectiveness of RNs and nurse practitioners in primary care remains [77]. Notably, economic evaluations of nursing interventions often do not consider or adequately capture the importance of patient-relevant outcomes (e.g., patient satisfaction, patient knowledge, treatment adherence and self-management, health-related quality of life, and patient self-reported physical, mental, and social functioning) [77] or primary care RN contributions to other domains of practice that contribute to optimal health outcomes [7, 77, 78]. In order to provide a more comprehensive economic evaluation, all elements of RN care provision within primary care need to be taken into account, such as context of practice (e.g., team-based settings, remuneration, nurse characteristics), scope of practice, and robust methodologies that employ adequate comparator groups.

While all studies met minimum quality thresholds to be included in this review, a number of methodological issues remain. Many studies in the review tend to be limited to outcomes involving direct patient care, therefore overlooking the multidimensional nature of primary care RN competencies that includes contributions towards other domains of practice, such as quality improvement, research, education, collaboration and partnership, and leadership activities, that also contribute to health and well-being of patients and families [7, 77, 78]. Additionally, there were only 12 studies that employed a design with a control/comparator group (the other studies were quasi-experimental/observational in nature). Choosing an appropriate comparator can present a challenge, as ‘usual care’ is often not well-defined and may be unique to a specific type or model of care (e.g., team-based care, nurse-led) or jurisdiction, making it difficult to apply results on a broader scale [77, 79]. Within the primary care setting, there are also many challenges associated with isolating and measuring the impact of individual health providers within the context of a team, such as the complex nature of roles and variability in practice settings. For example, many studies specific to primary care RNs focus on interdependent roles within broader healthcare teams, which often involves the shifting of work from one provider to another (e.g., physician to a RN) or care provided in collaboration with another team member [77, 79]. Additionally, studies must address role-specific considerations, such as differentiating between interdependent and independent activities within the primary care setting.

### Strengths and limitations

Strengths of this systematic review include the application of a comprehensive search strategy, use of the PRISMA checklist in planning and reporting, and appraisal using an established quality assessment tool (i.e., ICROMS). However, despite utilizing a comprehensive search strategy, it is possible that not all relevant studies were retrieved and included in this review. This review exclusively examined studies in which an intervention was delivered by a RN. In many cases, the RN designation was not stated or could not be clearly determined, therefore resulting in exclusion from the review. The lack of consistent terminology and available data regarding terminology used to describe RNs, or equivalent nursing titles, across countries limited the ability to include studies published in certain regions; however, we did attempt to compensate for the variation in terminology in our search mesh terms. Only studies published in the English language were included, which may limit generalizability to certain countries and exclude potential findings published in other languages. Furthermore, we identified four economic evaluations of RNs in primary care using the search strategy. Further research specifically targeting economic evaluations is needed to fully assess the cost implications of primary care RNs. Due to the limited number of high-quality randomized controlled trials, which provide the strongest level of evidence, the generalizability of findings from this study are limited. Similarly, the generalizability of the findings are further limited by the inclusion of a broad range of study designs, RN-led interventions (which were delivered independently by the RN or in collaboration with other providers and included different comparison/control groups), and outcome measures. Due to this diversity, meta-analysis was not possible and the findings should be interpreted with caution.

## Conclusions

Primary care RNs are increasingly becoming embedded into the core of interprofessional primary care teams [3, 4]. Overall, the findings suggest that primary care RNs impact the delivery of quality primary care, and that RN-led care may complement and potentially enhance primary care delivered by other primary care providers. RNs can provide appropriate, high quality primary care services, including but not limited to, medication management, patient triage, chronic disease prevention and management, treatment of acute illnesses/conditions, educational interventions, health promotion, and management interventions. Greater resources need to be directed towards evaluating the contribution of this unique role in primary care in order to optimize and strengthen the delivery of patient-focused care. Findings from this review can inform further integration and optimization of this role, are applicable to researchers and other stakeholders engaged in primary care interventions, and can assist with future evaluations and the development of more efficient primary care services. As increasing numbers of RNs are employed in primary care, more rigorous approaches to research employing robust study designs needs to be conducted to further understand the impact of RNs on care delivery and system outcomes in primary care.

## Supplementary Information

**Additional file 1.**

**Additional file 2.**

**Additional file 3.**

**Additional file 4.**

## Data Availability

All data generated or analysed during this study are included in this published article.

## Abbreviations

RN: Registered Nurse
JBI: Joanna Briggs Institute
PRISMA: Preferred Reporting Items for Systematic Reviews and Meta-Analysis
PROSPERO: Prospective Register of Systematic Reviews
ICROMS: Integrated Quality Criteria for Review of Multiple Study Designs
CI: Confidence Interval
OR: Odds Ratio
CPS: Clinical Pharmacy Specialist
MBS: Medicare Benefits Schedule
BMI: Body Mass Index
HbA1C: Hemoglobin A1C
QALY: Quality-Adjusted Life-Year
