# Supplementary material for: Effectiveness of registered nurses on system outcomes in primary care: a systematic review
Source: BMC Health Serv Res. 2022 Apr 4;22:440. doi: 10.1186/s12913-022-07662-7 (PMC8981870; doi:10.1186/s12913-022-07662-7)

**Supplementary File 1.** Adapted from the Nursing Role Effectiveness Model (Irvine et al., 1998; Lukewich et al., 2019)


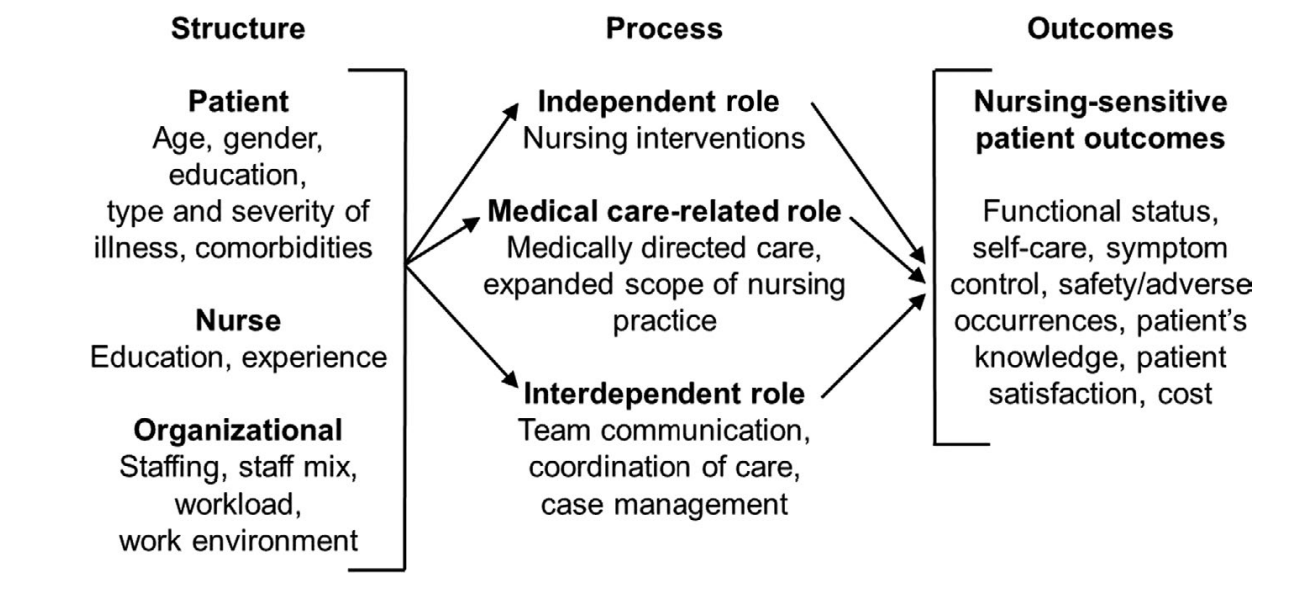

Supplement: Supplementary file 1 — Additional file 1. [file 12913_2022_7662_MOESM1_ESM.docx]
