# Supplementary material for: Effectiveness of registered nurses on system outcomes in primary care: a systematic review
Source: BMC Health Serv Res. 2022 Apr 4;22:440. doi: 10.1186/s12913-022-07662-7 (PMC8981870; doi:10.1186/s12913-022-07662-7)
Supplement: Supplementary file 2 — Additional file 2. [file 12913_2022_7662_MOESM2_ESM.docx]

**Supplementary File 2, Complete Search Strategy for Each Database**

**CINAHL Search (via EBSCO), conducted 5 January 2022**

| **#** | **Query** | **Results** |
| --- | --- | --- |
| S1 | ((TI nurs* OR TI RN*) N1 (TI "primary care" OR TI "primary health care" OR TI "primary healthcare" OR TI "family practice" OR TI "general practice" OR TI GP)) OR TI "office nurs*" OR TI "clinic nurs*" | 1,845 |
| S2 | (AB nurs* OR AB RN*) N0 (AB "primary care" OR AB "primary health care" OR AB "primary healthcare" OR AB "family practice" OR AB "general practice" OR AB GP) | 1,787 |
| S3 | (MM "Nurses" OR MM "Registered Nurses" OR MM "Office Nursing" OR MM "Community Health Nursing") AND (MM "Primary Health Care" OR MM "Family Practice" OR MM "Community Health Centers") | 2,072 |
| S4 | S1 OR S2 OR S3 | 4,851 |
| S5 | S1 OR S2 OR S3 (Limiters: English Language) | 4,512 |

| **#** | **Query** | **Results** |
| --- | --- | --- |
| S1 | ((TI nurs* OR TI RN*) N1 (TI "primary care" OR TI "primary health care" OR TI "primary healthcare" OR TI "family practice" OR TI "general practice" OR TI GP)) OR TI "office nurs*" OR TI "clinic nurs*" | 1,238 |
| S2 | (AB nurs* OR AB RN*) N0 (AB "primary care" OR AB "primary health care" OR AB "primary healthcare" OR AB "family practice" OR AB "general practice" OR AB GP) | 1,248 |
| S3 | MM "Primary Care Nursing" OR ((MM "Nurses" OR MM "Office Nursing" OR MM "Nurses, Community Health" OR MM "Community Health Nursing") AND (MM "Primary Health Care" OR MM "Family Practice" OR MM "Physicians' Offices" OR MM "Community Health Centers")) | 1,884 |
| S4 | S1 OR S2 OR S3 | 3,684 |
| S5 | S1 OR S2 OR S3 (Limiters: English Language) | 3,287 |

**MEDLINE Search (via EBSCO), conducted 5 January 2022**

**APA PsycInfo Search (via EBSCO), conducted 5 January 2022**

| **#** | **Query** | **Results** |
| --- | --- | --- |
| S1 | ((TI nurs* OR TI RN*) N1 (TI "primary care" OR TI "primary health care" OR TI "primary healthcare" OR TI "family practice" OR TI "general practice" OR TI GP)) OR TI "office nurs*" OR TI "clinic nurs*" | 241 |
| S2 | (AB nurs* OR AB RN*) N0 (AB "primary care" OR AB "primary health care" OR AB "primary healthcare" OR AB "family practice" OR AB "general practice" OR AB GP) | 352 |
| S3 | (MM "Nurses" OR MM "Nursing") AND MM "Primary Health Care" | 658 |
| S4 | S1 OR S2 OR S3 | 977 |
| S5 | S1 OR S2 OR S3 (Limiters: English Language) | 965 |

**Embase Search (via Embase.com), conducted 5 January 2022**

| **#** | **Query** | **Results** |
| --- | --- | --- |
| S1 | (((nurs* OR rn) NEAR/2 ('primary care' OR 'primary health care' OR 'primary healthcare' OR 'family practice' OR 'general practice' OR gp)):ti) OR 'office nurs*':ti OR 'clinic nurs*':ti | 1,406 |
| S2 | (((nurs* OR rn) NEAR/1 ('primary care' OR 'primary health care' OR 'primary healthcare' OR 'family practice' OR 'general practice' OR gp)):ab) OR 'office nurs*':ab OR 'clinic nurs*':ab | 2,392 |
| S3 | ('nurse'/mj OR 'registered nurse'/mj) AND ('primary health care'/mj OR 'primary medical care'/mj) OR 'primary nursing'/de | 762 |
| S4 | S1 OR S2 OR S3 | 4,006 |
| S5 | S1 OR S2 OR S3 (Limiters: English Language) | 3,687 |

**Gray Literature Searches**

**ProQuest Dissertations and Theses, conducted 5 January 2022**

ti(((nurs* OR RN*) NEAR/1 ("primary care" OR "primary health care" OR "primary healthcare" OR "family practice" OR "general practice" OR GP)) OR "office nurs*" OR "clinic nurs*" ) OR ab(((nurs* OR RN*) NEAR/0 ("primary care" OR "primary health care" OR "primary healthcare" OR "family practice" OR "general practice" OR GP)) OR "office nurs*" OR "clinic nurs*" )

**MedNar, conducted 5 January 2022**

Title: "primary care nursing" OR "primary care nurse" OR "primary care nurses" OR "primary healthcare nursing" OR "primary healthcare nurse" OR "primary healthcare nurses" OR "primary health care nursing" OR "primary health care nurse" OR "primary health care nurses" OR "family practice nursing" OR "family practice nurse" OR "family practice nurses" OR "general practice nursing" OR "general practice nurse" OR "general practice nurses" OR "office nursing" OR "office nurse" OR "office nurses" OR "clinic nursing" OR "clinic nurse" OR "clinic nurses"

**Websites Searched**

International Nursing Council - <https://www.icn.ch/>

Canadian Family Practice Nurses Association - <https://www.cfpna.ca/>

Community Health Nurses of Canada - <https://www.chnc.ca/en/>

Canadian Nurses Association - [https://www.cna-aiic.ca/en/](https://www.cna-aiic.ca/en/e)

Google Scholar - <https://scholar.google.ca/>
