# Supplementary material for: Effectiveness of registered nurses on system outcomes in primary care: a systematic review
Source: BMC Health Serv Res. 2022 Apr 4;22:440. doi: 10.1186/s12913-022-07662-7 (PMC8981870; doi:10.1186/s12913-022-07662-7)
Supplement: Supplementary file 4 — Additional file 4. [file 12913_2022_7662_MOESM4_ESM.docx]

**Supplementary File 4. List of Study Limitations (n=17)**

| **Author Name, Year** | **Limitations** |
| --- | --- |
| Aubert et al., 1998 | **ICROMS Score**: 25  **Study Design:** Randomized controlled trial  -Only one RN (certified in diabetes education) involved in study; difficult to determine if results would be generalizable  -Intervention group had fewer members of ethnic minority groups, more smokers, more insulin-treated patients, and a larger number of patients with type 1 diabetes  -Fairly high attrition rate (34% did not go to scheduled appointments; an additional 38 patients did not provide 12-month follow-up data). The majority of patients lost to follow-up were younger (18-44 years) and non-white  -Study involved implementation of an algorithm developed by researchers, the participation of physician advocates, and the RN to work closely with an endocrinologist and physician. It is difficult to discern whether the same results would be achieved under more ‘real world’ situations |
| Azariah et al., 2013 | **ICROMS Score**: 22  **Study Design:** Uncontrolled before-after  -Length of intervention was not indicated  -Study doesn’t contain a great deal of information regarding the nursing role or the actual intervention itself  -There was some contamination of laboratory data; reports also contained data from three practices in the same region not included in the research project due to the fact that they used the same laboratory reference codes  -No documentation was kept regarding how many people were offered testing or how many declined testing, therefore test uptake could not be measured  -Approaches to collecting specimens for testing shifted during the pilot project to offering self-collected vaginal swabs to females, which may have influenced uptake |
| Bellary et al., 2008 | **ICROMS Score**: 24  **Study Design:** Cluster randomized controlled trial  -More patients in the control group than in the intervention group were ex-smokers; more patients in the intervention group were being treated with statins  -RNs had protected time to run a research diabetes clinic and the support of diabetes specialist community nurses; may not be generalizable to ‘real world’ care provision  -Study sample contained patients who were at a high cardiovascular risk, target HbA1c values may have been too difficult to achieve in this particular group  -Despite study protocol stating that all patients should be prescribed statins, only two-thirds were actually receiving them, which may have influenced the results  -Intervention contained many complex components (including care by link workers and a community nurse specialized in diabetes), it is difficult to assess the relative contributions of each individual component, specifically, the extent to which the RN role had on outcomes  -For patients with missing data, the authors employed the ‘last observation carried forward’ method, which is a method with known weaknesses |
| Cherkin et al., 1996 | **ICROMS Score**: 24  **Study Design:** Randomized controlled trial  -Study is somewhat dated (conducted from 1992-1993); changes may have taken place since then regarding methods of patient-centered educational interventions  -RNs in study had at least 20 years of clinical experience; this may not be generalizable to RNs with less experience.  -Attrition rate was fairly high (only 34% of initial sample had at least one week follow-up data).  -Rather than standard randomization, ‘pre-consent randomization’ was chosen  -Patient satisfaction ratings were obtained only at baseline and at a 1-week follow-up evaluation  -All outcomes were assessed by self-report measures; patients in the RN follow-up group may have felt compelled to embellish their level of physical activity/adherence to recommendations  -Participant sample was fairly uniform demographically (predominantly white, highly educated, employed, generally physically active); may not be generalizable to a wider population |
| Daly et al., 2020 | **ICROMS Score:** 21  **Study Design:** Observational; cross-sectional survey  -Lists used to identify and recruit RNs differed between recruitment periods; it is possible that characteristics of RNs differed as a result of this (although the response rate for both surveys was sufficiently high and the selection process was done through random sampling)  -Study relies heavily on self-report data  -Weighting the sampled patients could potentially under- or over-inflate differences between patient survey groups, as this is based on the assumption that the same nurse provided the same care to all patients consulted. It is possible that patients not surveyed differed in terms of demographics and biophysical characteristics than those surveyed by the same nurse  -Patient records of previous examinations were incomplete  -Study employed an observational design; there was no control group comparison or way to account for potential confounders |
| Farford et al., 2020 | **ICROMS Score:** 19  **Study Design:** Observational; retrospective chart review  -Study focused only on screening/preventative services delivered to Medicare beneficiaries (reserved for individuals 65 years or older or younger people with certain disabilities). Additionally, there is a likelihood that participants in this study were more likely to be engaged in their own health; this may not be generalizable to the rest of the population  -Only eight preventative services were examined in this study; results may differ for screening services that were not examined  -Certain screening or preventative services may have been provided outside the study clinic and not appropriately documented in the EMR, and therefore not accounted for in the chart review  -It is possible that some patients may not have been candidates for certain services and were inaccurately assumed not to have received the service  -Socioeconomic status, race, and ethnicity were not taken into account or documented in the study (a known factor related to care access), which limits the ability to evaluate how RN-led annual wellness visits influences preventative care in the wider patient population  -Study employed a retrospective chart-review design; there was no control group comparison or way to account for potential confounders |
| Faulkner et al., 2016 | **ICROMS Score**: 21  **Study Design:** Cohort study using longitudinal data from a previously conducted randomized controlled trial  -Participants were required to have a mobile phone and the knowledge/ability to send and receive text messages; this may disproportionately affect patients from certain demographics (e.g., lower socioeconomic statuses, older patients, etc.)  -Relatively low uptake among practices approached (31%); sample of participating practices was not fully representative of areas of deprivation across England  -While the 4-week smoking outcome was biochemically verified, the 8-week and 6-month outcomes were measured by self-report only  -Study did not report on whether questionnaire was a validated tool or how it was developed  -Study should have followed up with participants beyond 6 months and had their abstinence biochemically verified in order to confirm long-term smoking abstinence  -Data was collected from patients 8 weeks after the initial consultation, increasing the risk of recall bias or erroneous recall  -The type smoking cessation advisor seen by participants was not determined at random. While participants in each group had similar characteristics, there may have been unmeasured patient or advisor confounders that impacted findings |
| Gallagher et al., 1998 | **ICROMS Score**: 16; 22*  **Study Design:** Observational (cross-sectional) and uncontrolled before-after  -Intervention was carried out by only one RN who had 15 years of clinical experience and was familiar with and trained in conducting telephone consultations; may not be representative  -Some data was excluded due to poor information capture  -Postal questionnaire assessing patient satisfaction was only sent to patients who had received telephone advice from an RN, limiting the ability to make comparisons  -Physician workload reduction could be attributed to the season (summer months, “holiday period”), and thus a decline in overall patients; analysis compared workload during this time with the three months prior, rather than during the same season of the previous year. The period of data collection was too short to reveal diurnal and seasonal variations in acute illness workload  -There was a delay of 10 months in conducting the postal survey, which may affect the validity of the questionnaire findings and present issues with recall |
| Harris et al., 2015 | **ICROMS Score**: 28  **Study Design:** Cluster randomized controlled trial  -Study included a narrow age range (60-75 years) and may not be generalizable to a broader sample  -Participants in the intervention group were slightly older, less educated, more likely to be overweight/obese, more likely to have chronic diseases/disabilities, and had a slightly lower baseline step-count than those in the control group  -Neither the patients themselves nor the researchers were blinded to the group allocation  -Some patients were randomized without meeting the eligibility cut-off for recorded activity (≥ 5 days of ≥ 600 minutes); additionally, 12 were incorrectly randomized  -Not all participants in the intervention group attended all four nurse counseling sessions  -Affluent, non-ethnically diverse socio-economic areas were overrepresented in the sample  -Intervention was comprised of multiple components, making it difficult to determine which components were contributing to outcomes  -Patients may have been more motivated to engage in physical activity given that they knew they were being monitored (i.e., Hawthorne effect); results may be less in ‘real world’ scenarios |
| Harris et al., 2017 | **ICROMS Score**: 26  **Study Design:** Cluster randomized controlled trial  -Participants, nurses and researchers were unmasked to intervention allocation  -The RN-supported group had a slightly higher average daily step-count and minutes spent weekly in MVPA in bouts of ≥ 10 minutes at baseline than the other comparator groups  -Not all participants in the intervention group attended all three sessions  -Patients may have been more motivated to engage in physical activity given that they knew they were being monitored (i.e., Hawthorne effect); results may be less in ‘real world’ scenarios  -Recruitment rate was low (10%), raising issues of generalizability  -At baseline, only 21% of patients achieved guidelines based on accelerometry but were not excluded |
| Iles et al., 2014 | **ICROMS Score**: 22  **Study Design:** Randomized controlled trial; cost-analysis  -There were small sample sizes for each chronic disease, as well as a slightly higher proportion of patients with diabetes in two of the general practices  -Study only contained 3 practice locations, which may limit generalizability  -Cost analysis is measured based on total MSB charges; however, this method tends to be somewhat limited in capturing the full extent of healthcare associated costs  -Difficult to determine what caused the increased workload during the intervention period, this cannot be attributed to quality of care per se  -There is no follow-up or long-term data to predict changes to cost-effectiveness over time  -It is unknown whether the additional costs would result in better long-term health outcomes |
| Karnon et al., 2013 | **ICROMS Score**: 22  **Study Design:** Observational; risk-adjusted cost-effectiveness analysis  -Study was specifically looking at the role of primary care RNs in the practice setting in general; level of RN involvement in the care of individual patients included in the study itself was not examined  -BMI was used as the main measurement, which is a somewhat flawed method of assessing body fat  -Study employed an observational design; there was no control group comparison or way to account for potential confounders  -Uncertainty around cost-effectiveness, given that only 23% of all patients received inpatient care and the mean differences were driven by a few high-cost individuals  -Study would have benefitted from follow-up component to measure sustained differences over time  -Estimated quality-adjusted life year gain was based on the assumption that weight loss at 12 months would be regained over the following two years, which may not be the case  -Sample size was relatively small (n=175 across comparator groups)  -Study may have been subject to selection bias  -Differences were observed in the extent to which GPs recorded patient details (e.g., BMI) causing potential participants to be excluded and the observed effectiveness of nurse involvement in the high-level model to be underestimated  -There is some arbitrariness and uncertainty around the criteria used to allocate practices to the different models, as the categorization is based only on the RN’s subjective response. |
| Katz et al., 2004 | **ICROMS Score**: 27  **Study Design:** Secondary analysis of data from a randomized controlled trial  -Secondary analysis of data  -Patients were interviewed in-person by study personnel immediately after the appointment and therefore may be motivated to give more positive responses  -Medical assistants in the study had significantly less experience than RNs, and in general, LPNs have less training in patient education and counseling than RNs, making for a less equivalent comparison |
| Low et al., 2005 | **ICROMS Score**: 27  **Study Design:** Randomized controlled trial  -RNs carried out initial partner notification at the time of diagnosis but did not follow-up index cases  -RNs were not randomly selected, but were nominated by participating practices; potentially only the most experienced nurses could have been nominated and thus not representative  -Training regarding management of chlamydia was offered; it was not stated if participating RNs had previous experience working with sexually transmitted infection treatment and management  -Comparison of the effectiveness between groups was influenced by the fact that a third of patients referred to the genitourinary medicine clinic (usual care) did not attend  -Low overall uptake of home-based screening reduced the number of eligible participants  -Study had a 30% default rate (patients not attending clinic) |
| Moher et al., 2001 | **ICROMS Score**: 26  **Study Design:** Pragmatic, unblinded cluster randomized controlled trial comparing three intervention arms  -Primary care RNs received education on how to implement the specific clinic protocol and had ongoing support from the trial’s nurse facilitator; it is difficult to determine whether or not the same results would be generalizable to ‘usual’ primary care  -Information about antiplatelet treatment was confined to prescriptions, as the study did not collect records of patient self-medication  -The practices participating in the study tended to be large, with good pre-existing nursing support (as opposed to many of the non-participating practices that had minimal nursing support); therefore, results may not be generalizable  -Trial took place in the context of a health authority audit initiative relating to patients with coronary heart disease; the observed intervention effect may have been greater otherwise |
| O’Neill et al., 2014 | **ICROMS Score**: 18  **Study Design:** Observational; non-randomized retrospective comparison of a natural experiment  -RNs are involved in the team composition of both comparison groups; it is difficult to determine the extent to which outcomes can be specifically attributed to the RN role itself  -Intervention occurred over a very short time span (~1 month)  -There was a greater proportion of white patients in the physician group than the comparator  -Since only patients with hypertension were included, similar trends between both groups may have simply been a result of regression towards the mean  -Study is exploratory in design, therefore not designed to fully assess effectiveness  -The study setting (Veteran’s Affairs medical center) may not be generalizable to a standard patient population, given its primarily male patient population  -Study used previously documented, unstandardized data from a variety of clinicians; preventing the ability to control for confounding variables  -Patients who were non-adherent with treatment or follow-up were excluded from the analysis and information surrounding excluded patients was not collected, likely affecting outcomes |
| Plummer et al., 2000 | **ICROMS Score**: 19  **Study Design:** Observational  -Study did not contain a control group  -Study does not elaborate on reason for the patient visit; the RNs’ ability to read emotional distress in a patient may differ based on patient affect/characteristics/comfort, etc.  -There were a number of confounding factors not taken into consideration (e.g., familiarity between patient and RN, appointment length, RN’s interest in emotional difficulties, patient characteristics)  -The only measure of patient psychological distress was self-report; psychiatric diagnoses of patients were not available to the research team  -Patients may downplay or overinflate their degree of emotional distress or attempt to provide answers that are more socially acceptable  -Patient views of practice nurse as an appropriate professional with whom to share emotional problems were not gathered as part of this study. Psychological distress may not have been evident during the patient-RN encounter |
| **Mixed methods study consisting of multiple designs; separate ICROMS quality appraisal scores were generated for each study type; RN – registered nurse; HbA1C – hemoglobin A1C; MVPA – moderate to vigorous physical activity; MSB – Medicare Benefits Schedule; BMI – body mass index; GP – general practitioner; LPN – licensed practice nurse* | |
